# A single-patient-use ECG system for cardiothoracic surgery admissions in the UK: A cost-consequence analysis

**DOI:** 10.3389/fpubh.2023.1027977

**Published:** 2023-03-30

**Authors:** Rhodri Saunders, Marco Caterino, Pranav Somaiya

**Affiliations:** ^1^Coreva Scientific GmbH & Co KG, Königswinter, Nordrhein-Westfalen, Germany; ^2^Department of Vascular Surgery, Barts Health NHS Trust, London, United Kingdom

**Keywords:** CABG, thoracic surgery, single-patient, ECG, cost-consequence analysis, NHS, costs

## Abstract

**Background:**

Deep sternal wound infections (DSWI) are severe complications in up to 1.36% of coronary artery bypass grafting (CABG) procedures in the United Kingdom. Each event adds between £4,000 and £11,000 in healthcare costs, owing primarily to prolonged hospitalisations. ECG devices have been shown to convey infection throughout perioperative CABG. On the other hand, single-patient ECG devices (spECG) can effectively reduce the incidence of surgical site infections (SSI), including DSWI, but no assessment of spECG impact in NHS cardiac units has been conducted.

**Methods:**

To estimate the impact of spECG on NHS cardiac units, we conducted a cost-consequence analysis modeling the CABG care pathway in the United Kingdom using Simul8 software for a probabilistic, individual-patient simulation. The simulation time was 1 year, with each patient followed from admission through 30 days post-discharge. The base case simulation mirrors the cardiac unit of Bart Health NHS Trust, London. A total of 2,183 patients are generated with demographic and clinical attributes from probabilistic distributions informed by hospital-specific inputs from NHS Digital Data. The Brompton Harefield Infection Score (BHIS) is allocated to gauge the risk of SSI. Results are averaged across 50 independent and randomly seeded iterations.

**Results:**

Simulation results indicate a base-case savings of £388 per patient, determined by the incidence of infections rather than the number of CABG procedures. In the base-case simulation, the mean cost of care with rECG was £13,096, whereas the mean cost with spECG was £12,708, resulting in a cost saving of £388 (2021 GBP). The simulation yielded an overall 8.6% SSI incidence rECG, whereas the incidence of SSIs with spECG was 6.9%. The model was most sensitive to changes in general ward and ICU costs, and infection incidence was a stronger predictor of potential per-patient savings than annual CABG volume.

**Conclusion:**

Single-patient ECG is a sustainable and effective alternative to reusable ECG cables and lead wires in terms of patient safety and resource allocation.

## 1. Introduction

Coronary artery bypass grafting (CABG) is the most common cardiac surgery worldwide, generally executed *via* median sternotomy (1, 2). Deep sternal wound infections (DSWIs) are rare yet severe complications in 0.5 to 6.0% of median sternotomy procedures, according to varying estimates (3, 4). DSWIs are challenging to treat and frequently escalate into complications with a poor prognosis, substantially longer hospitalisations, and a 10 to 50% mortality rate (5–7). The United Kingdom‘s National Institute for Cardiovascular Outcomes Research (NICOR) reported the average countrywide DSWI rate following CABG at 0.3% as of 2019 (8). However, the report elaborates on the heterogeneity of these figures across healthcare structures, with larger hospitals providing more confident rates, as high as 1.36% (8). The conceivable consequences for the healthcare system have been quantified between ~4,200 and ~11,000 Great British Pounds (GBP, £) in England, owing primarily to prolonged hospitalisations (or length of stay—LOS), between 9 and 23 days, depending on the primary procedure and complications (9–12).

Initiatives undertaken to improve the DSWI risk assessment and management have deemed certain “minor” aspects within the operating theater essential in preventing DSWI (9, 10), in particular wary perioperative prophylaxis of ECG devices, known vectors of infection (11). The complex surfaces and grooves of equipment, the extra workload on ward staff, and inconsistent protocols make sanitizing reusable ECG (rECG) monitoring wires challenging and often ineffective, leading to an increased risk of cross-contamination (13–15). Single-patient ECG (spECG) components have been shown to reduce the likelihood of surgical site infections (SSIs) in several studies (12, 15–17); The National Institute for Health and Care Excellence (NICE) assessed spECG technology in 2019 (18). Although acknowledging the innovative nature of the technology and its potential beneficial impact, NICE did not envisage the implementation nor resolve the clinical and monetary implications from the National Health Service (NHS) perspective (18).

To assess the potential impact of spECG on NHS cardiac units, we utilized a modeling approach to simulate the CABG care pathway at the individual-patient level. Our model followed patients for one year, from admission to 30 days after discharge, and compared costs and outcomes between spECG and the standard of care rECG, using publicly available NHS Digital Data from the UK's Health and Social Care Information Center.

The modeling approach was chosen due to several factors, including the lack of systematic implementation of spECG in the NHS, the National Institute for Health and Care Excellence's (NICE) call for UK-specific economic analyses (18), and the strain on ICUs caused by the COVID-19 outbreak, which made randomized clinical trials and empirical studies impractical. Our model provides a preliminary assessment of the potential impact of spECG on costs and outcomes in NHS cardiac care to inform future research and decision-making.

## 2. Methods

A cost-consequence analysis was designed and performed abiding by the good practice guidance from the International Society for Pharmacoeconomics and Outcomes Research (ISPOR) (19), The National Institute for Health and Care Excellence (NICE) (20), and the European Network for Health Technology Assessment (EUnetHTA) (21). The reporting is aligned with the Consolidated Health Economic Evaluation Reporting Standards (CHEERS) (22). The model takes the NHS hospital payers' perspective with costs reported in 2021 GBP.

### 2.1. Data source

Public data from the NHS Digital (23), UK's governmental agency responsible for providing information, data, and IT systems to support health and social care services in England, was used to inform the model of the costs, epidemiology, outcomes of different treatment options for coronary artery bypass grafting (CABG), and hospital-specific statistics on yearly procedures, patient demographics, etc. (Table 1). Specifically, the data used in this study pertains to procedures with codes K401–K404 and K453, which refer to saphenous vein graft replacement of coronary arteries and anastomosis of the mammary artery to the left anterior descending coronary artery, respectively, according to the OPCS-4.9 classification (32). Missing cost data were retrieved from Public Health Scotland, under the assumption that these would not significantly diverge from costs in NHS England. Other parameters were obtained from Barts Health NHS Trust, London, UK, and from a structured search of PubMed and EconLit conducted in August 2021. All key inputs are provided, with their reference sources in Table 1.

**Table 1:** Input parameters for the base-case simulation.

**Parameter**	**Value**	**SD**	**Distribution**	**Unit**	**References**
CABG	2,275	N/a	N/a	*n*/year	Barts data, NHS Digital (23)
Age	68.0	3.0	Normal	Year	UKHSA (24)
Sex	81.7	4.0	Binomial	% male	UKHSA (24)
BMI	28.6	4.5	Normal	kg/m^2^	UKHSA (24, 25)
Diabetes	23.7	0.0	Binomial	%	(25)
HbA1c >7.5	7.5	0.0	Bernoulli	%	(26)
LVEF < 45%	5.0	0.0	Bernoulli	%	Barts data, NHS Digital (23)
Requires MV	40.0	2.0	Normal	%	Barts data, NHS Digital (23)
MV time	8.0	2.0	Normal	Hour	Barts data, NHS Digital (23)
ECG time	24.0	4.0	Normal	Hour	Barts data, NHS Digital (23)
ICU LOS	1.0	0.2	Triangular	Day	Barts data, NHS Digital (23)
GW LOS	9.7	1.2	Triangular	Day	Barts data, NHS Digital (23)
Emergency surgery	2.0	1.0	Binomial	% CABG	UKHSA (24), NHS Digital (23)
spECG	[0.0 | 100.0]	N/a	N/a	% CABG	assumption
rECGs reuses	100.0	N/a	N/a	n	Cardinal Health Inc.
**SSI incidence and consequences**					
Additional LOS, deep SSI	24.6	2.5	Normal	Day	Barts data, NHS Digital (23)
Additional LOS, SSI	8.0	0.8	Normal	Day	UKHSA (24)
After time period	11.6	1.2	Normal	Day	Barts data, NHS Digital (23)
DSWI incidence	20.0	2.0	Normal	% SSI	UKHSA (24)
Readmission LOS	12.6	1.3	Normal	Day	Barts data, NHS Digital (23)
SSI incidence	3.96	0.0	N/a	%	Barts data, NHS Digital (23)
**Resource cost**					
Consultant	122.0	3.5	Normal	GBP/hour	PSSRU 2021 (27)
DSWI	12.0	2.0	Normal	GBP/day	PSSRU 2021 (27)
GW	28.0	2.8	Normal	GBP/hour	PHS, D025_2019 (28)
ICU	82.0	4.0	Normal	GBP/hour	PHS, R040X_2019 (29)
Mediastinitis	18.0	2.0	Normal	GBP/day	PSSRU 2021 (27)
MV	40.0	12.0	Normal	GBP/day	Barts data, NHS Digital (23)
Nurse	44.0	4.4	Normal	GBP/hour	PSSRU 2021 (27)
Outpatient SSI treatment	40.0	4.0	Normal	GBP/visit	PSSRU 2021 (27)
PACU	82.0	8.2	Normal	GBP/hour	Assumed as ICU
rECG cable	60.0	4.0	Normal	GBP/unit	Barts data, NHS Digital (23)
rECG decontamination	0.2	0.1	Normal	GBP/unit	Cardinal Health Inc.
rECG lead	80.0	5.0	Normal	GBP/unit	Barts data, NHS Digital (23)
spECG (Kendall DL™)	12.7	1.3	Normal	GBP/unit	Cardinal Health Inc.
SSI treatment	5.0	1.0	Normal	GBP/day	PSSRU 2021 (27)
Surgery	594.0	59.4	Normal	GBP/hour	PHS, R142X_2019 (29)
**Staff resources**					
Consultant handover time	5.0	1.0	Normal	Minute	Barts data, NHS Digital (23)
Consultant ICU examination time	5.0	1.0	Normal	Minute	Barts data, NHS Digital (23)
Nurse check time	1.0	1.0	Normal	Minute	Barts data, NHS Digital (23 )
Nurse discharge time	10.0	1.0	Normal	Minute	Barts data, NHS Digital (23)
Nurse handover time	5.0	1.0	Normal	Minute	Barts data, NHS Digital (23)
Nurse ICU examination time	10.0	1.0	Normal	Minute	Barts data, NHS Digital (23)
Surgical preparation	45.0	5.0	Normal	Minute	Barts data, NHS Digital (23)
**Mortality**					
Surgery related mortality	1.5	0.0	N/a	%	(30)
With DSWI	0.8	0.0	N/a	%	(30)
**SSI risk**					
BHIS [0, 1]	0.57	0.0	N/a	N/a	(26)
BHIS [2, 3]	1.32	0.0	N/a	N/a	(26)
BHIS ≥4	3.51	0.0	N/a	N/a	(26)
Relative SSI risk with spECG	0.76	0.0	N/a	N/a	(13, 15, 16, 31)
**Model timings**					
Surgery	2.0	0.2	Normal	Hour	Barts data, NHS Digital (23)
GW check	4.0	0.0	N/a	Hour	Barts data, NHS Digital (23)
ICU check	2.0	0.0	N/a	Hour	Barts data, NHS Digital (23)
Initial ICU time	4.0	0.2	Normal	Hour	Barts data, NHS Digital (23)
PACU time	2.0	0.2	Normal	Hour	Barts data, NHS Digital (23)

Costs are given in 2021 GBP. Barts, Barts Health NHS Trust, London, UK; BHIS, Brompton and Harefield Infection Score; BMI, body mass index; CABG, coronary artery bypass grafting; DSWI, deep sternal wound infection; ECG, electrocardiography; GW, general ward; HbA1c, glycated hemoglobin A1c; ICU, intensive care unit; LOS, length of stay; LVEF, ejection fraction; MV, mechanical ventilation; N/A, not applicable; NHS, National Health Service; PACU, post-anesthesia care unit; PHS, Public Health Scotland; PSSRU, Personal Social Services Research Unit; R025, PHS Scotland data series on board level aggregate of hospital running cost; R040X, PHS Scotland data series speciality costs and activity-inpatients in long stay specialities, by speciality; R142X, PHS Scotland data series on average theater running costs and usage by speciality and by board, from Public Health Scotland; rEGC, reusable ECG; spECG, single-patient ECG; UKHSA, United Kingdom Health Safety Agency.

### 2.2. Model design and structure

**Figure 1:**
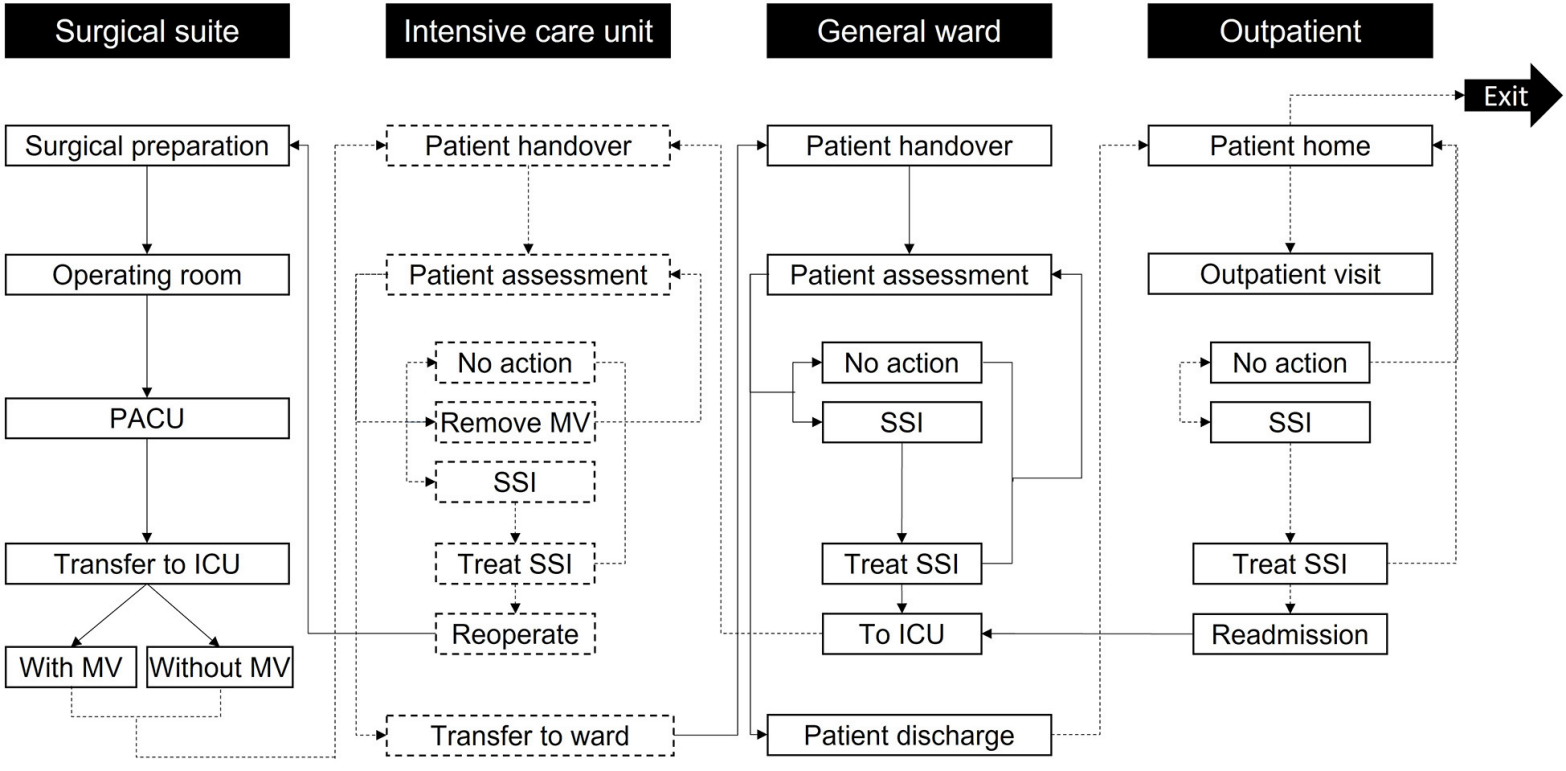
Model care settings and activities. Patients enter the simulation at surgical preparation and then progress through the care pathway until exiting the simulation after 30 days in the outpatient setting. Reoperation is only accessed if the patient develops mediastinitis; otherwise, other non-operative treatments are used in the “Treat SSI” activities. The labels in the black boxes indicate the care setting, while the labels in the white boxes show activities in each care setting. To help differentiate the activities per setting, the boxes referring to the surgical suite and GW activities are marked with solid black lines, while the boxes concerning the ICU and outpatient use activities are marked with dashed black lines. Arrows linking activities follow the same principle based on the activity they go to. ICU, Intensive Care Unit; MV, Mechanical ventilation; PACU, Post-Anesthesia Care Unit; SSI, Surgical-site infection.

The CABG care pathway (Figure 1) to estimate the impact on costs and outcomes of spECG monitoring in cardiac units was designed from PS's clinical experience and hospital management perspective. The model is a probabilistic, individual-patient, discrete-event simulation as defined in Brennan et al. (33). The pathway simulation was developed in Simul8 (Simul8 Corporation, Boston, MA, USA) based on our published Markov model (34). The model progresses in one-minute increments, returning 1,440 assessments per patient per simulated day. Each simulation and each patient are seeded entities, such that the “same” patients are used for generating estimates during the same iteration with spECG and rECG. To address the intrinsic stochastic uncertainty (35), the base-case simulation is iterated 50 times on the same seeded population, helping to ensure robustness and precision in estimating the model's outputs by accounting for the probabilistic nature of simulation runs.

Based on user input, the model generates and simulates as many unique patients as necessary to represent varied cardiac units. Each patient is assigned age, sex, Body Mass Index (BMI), diabetes (hemoglobin HbA1c >7.5%), and left ventricular ejection fraction (LVEF, <45%) at random from normal (age and BMI) or binomial (sex, diabetes, HbA1c, and LVEF) distributions described by mean and standard deviation (Table 1). These parameters are used to assign a Brompton Harefield Infection Score (BHIS) for adjusting the risk of SSI (36). In addition, the need for postoperative mechanical ventilation is assigned from a binomial distribution. The time on mechanical ventilation, the time in the intensive care unit (ICU), and time on the general ward (GW) are instead simulated per patient, drawing on normal distributions with a 10% standard deviation. A comprehensive list of inputs is found in Table 1.

### 2.3. Care pathway

**Figure 2:**
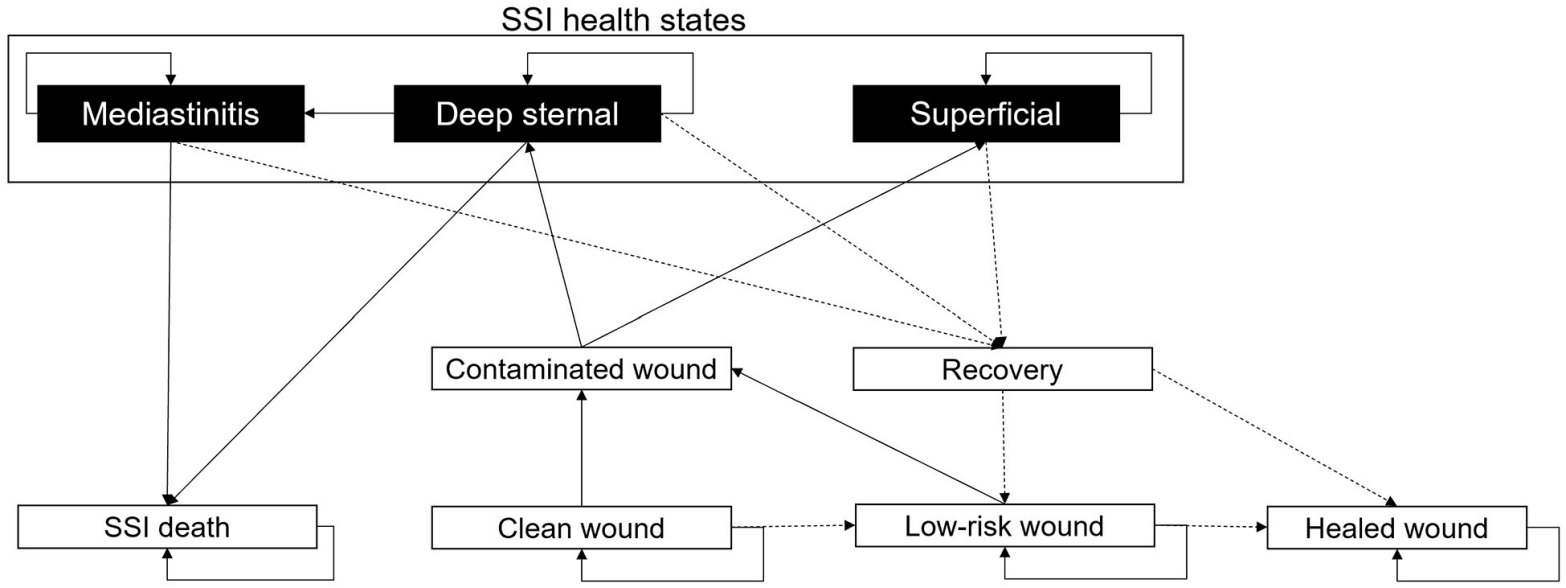
Markov model describing the development of SSIs. All patients are assigned a “Clean wound” following CABG surgery. From here, they have the chance to maintain a “Clean wound,” develop a “Contaminated wound,” or continue healing to a “Low-risk wound.” If the patient develops a “Contaminated wound,” then there is the chance of this being “Superficial” or “Deep-sternal.” A “Deep-sternal” infection can develop into “Mediastinitis.” “Deep-sternal” infection and “Mediastinitis” can lead to patient death, an “SSI death.” From any SSI health state, a patient's wound infection can recover with treatment; upon “Recovery,” the wound may be either considered a “Low-risk wound” or a “Healed wound.” From a “Healed wound,” no SSI will develop. A “Low-risk wound” may heal to a “Healed wound” or return to “Contaminated wound.” All patients discharged from hospital will have a “Low-risk wound.” In the diagram, solid black arrows indicate wound stability or infection progression, and dashed black arrows represent healing.

Patients proceed through “locations” within the care pathway (Figure 1) and remain therein for periods assigned from distributions in Table 1 through non-independent time points. Health state transitions proceed through a Markov model (Figure 2) relevant to SSIs, with each patient assumed to exit CABG surgery with a “clean wound.” State-transition probabilities are calculated relative to the iterations per day and the patient's individual risk (BHIS). The model accounts for the surgical suite, the ICU, the GW, and the outpatient settings (Figure 1). Patients scheduled for CABG enter the simulation in “Surgical preparation” and progress to the “Operating room” and the “post-anesthesia care unit” (PACU). Patients are then transferred to the “ICU,” either on or off mechanical ventilation (MV), assessed for SSI by a nurse at set intervals, and evaluated for discharge onto the “General ward.” In the eventuality of SSI, a consultant evaluates the SSI as superficial, deep sternal, or mediastinal and accordingly assigns appropriate treatment. Upon developing mediastinitis, patients may be treated surgically (reoperation) or non-surgically. Patients are only transferred to the GW if they have no SSI and are not on MV.

As in the ICU, on the GW, patients undergo regular assessments by nursing staff. If an SSI is detected, a consultant assigns appropriate treatment. Patients are redirected to the ICU in the event of DSWI or mediastinitis. Superficial SSIs are treated on the GW. Patients are discharged upon completion of their hospital stay. Post-discharge SSIs (up to 30 days) are assessed in the “Outpatient” setting and treated at home if superficial. DSWI and mediastinitis result in readmission to the ICU.

After surgery, every patient is assumed to have a “Clean wound” (Figure 2). The probability of the “Clean wound” becoming contaminated is dependent on user inputs for the SSI incidence (the percentage of patients experiencing an SSI, Table 1, and the number of days on which this SSI incidence was recorded (SSI^Days^). The equation for the overall SSI probability per minute is given by $1-e^{\left(\frac{-\ln\left[1-SSI_{\%}\right]}{\ln\left[1-SSI_{day}\times 1,440\right]}\right)}$, adjusted by 0.76 relative risk for the spECG arm (13, 15, 16, 31). For a “Low-risk wound,” the probability of developing a “Contaminated wound” is 0.25 times (one quarter) that of a “Clean wound.” A “Low-risk wound” transitions to a “Healed wound” after 30 days. “Superficial” SSIs, “Deep sternal” infections, and “Mediastinitis” that do not result in patient death transition to recovery after a user-inputted number of days.

### 2.4. Model inputs

The NHS Digital Data (23) for K401-K404 (saphenous vein graft replacement of coronary arteries) and K453 (anastomosis of the mammary artery to the left anterior descending coronary artery) procedures were used to input the model with cardiac unit size, number of CABG per year, patient demographics and clinical characteristics, SSI and DSWI incidence, requirement for MV, timings (on ward and ICU/PACU check intervals and duration), staff resources and costs (Table 1). Other costs were taken from NHS England national reports and the Personal Social Services Research Unit (PSSRU) (27). Cost inputs unavailable for England were sourced from the Information Services Division (ISD) of NHS Scotland (28, 29). The relative risk of SSI with spECG (0.76) was informed by a structured literature search (13, 15, 16, 31).

### 2.5. Costs and consequences

The outcomes considered in this cost-consequence analysis were care costs, LOS, and SSI events. Costs were collected by care setting (surgical, ICU, GW, and outpatient) and ECG monitoring costs. The overall mean cost per patient was calculated at the end of the simulation. The consequences considered in this model were LOS in ICU, LOS on GW (both reported in total and mean days per patient), and SSI events. The SSI events were detailed as superficial or DSWI (including mediastinitis). The total cost of care and the potential savings ascribable to spECG were also reported.

### 2.6. Sensitivity analysis

**Figure 3:**
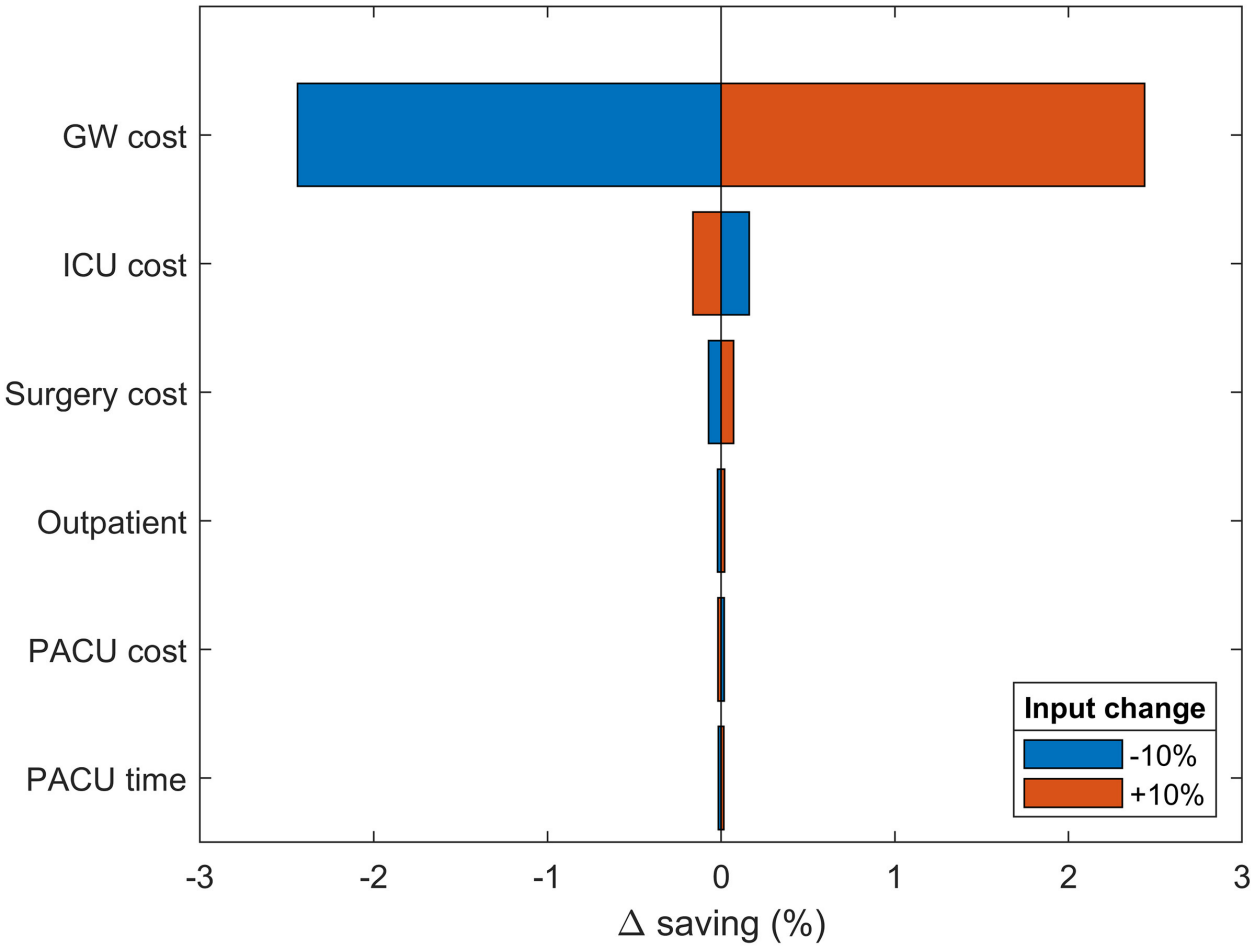
One-way sensitivity analysis. Changes in modeled savings upon ±10% variation in most impactful input parameters. The results are provided as the percentage deviation from the base case savings. Positive values favor spECG. Other inputs had irrelevant impact on savings and were therefore omitted.

We performed a semi-probabilistic sensitivity analysis to comprehensively assess the impact of changes in the mean input values on the model output (35). Mean input values for all model inputs were changed by an arbitrary ±10% from the base case while retaining specific probability distribution and standard deviation. Each sensitivity simulation is, therefore, a probabilistic simulation of each patient's progression through the model, averaged across 50 independent, seeded iterations to ensure robustness and precision (as with the base case). The same rationale was adopted for the semi-probabilistic three-way sensitivity analysis on the expected savings as a function of CABG/year and SSI incidence. The sensitivity results are reported as the percentage of deviation from base-case savings (Figure 3). A positive delta reflects larger predicted savings.

**Figure 4:**
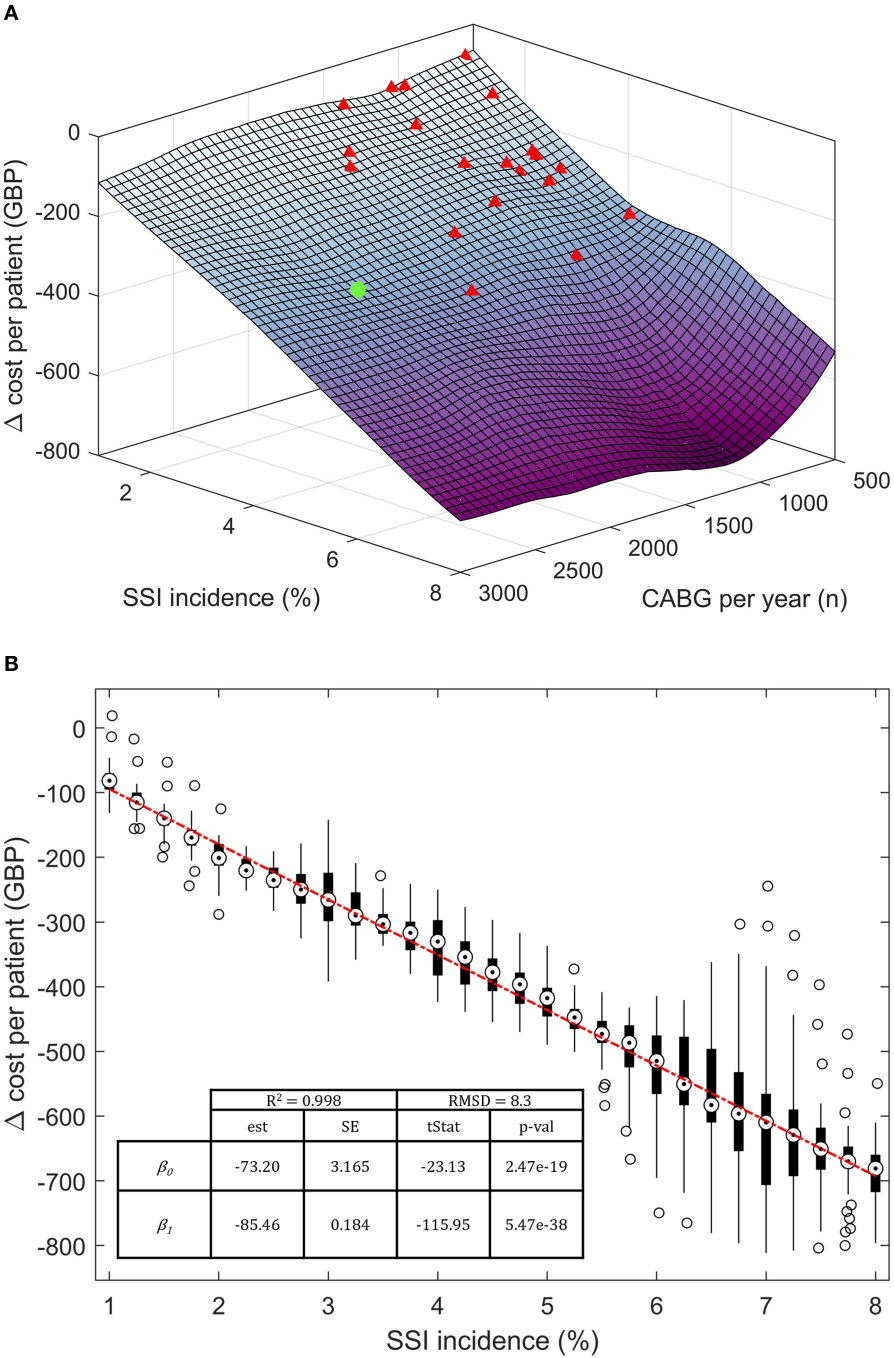
Three-way, discrete-point response saving response scenario. **(A)** Three-dimensional saving per patient response surface at varying SSI incidence and number of patients per year. **(B)** Bivariate regression on Δ cost per patient on the SSI incidence. Target markers are the median point of each cost vector. The boxes extend between the 25th and 75th percentiles. Hollow circles represent >2σ outliers. The base case is noted as a green dot; other NHS cardiac units are represented as red triangles. β0, intercept; β1, regression coefficient; est, estimates; SE, standard error, tStat, t-statistics; RMSD, root-mean-square deviation; *R*^2^, coefficient of determination.

The model response to SSI incidence and cardiac unit size (CABGs per year) was further investigated in a semi-probabilistic three-way, discrete-point analysis (Figure 4B). The SSI incidence interval was arbitrarily drawn around the base-case value, ranging between 1 and 8%. Incidence rates <1% were excluded due to surging inaccuracy in estimating the cost per patient, while rates >8% were deemed implausible. Similar logic led to the exclusion of structures with <500 CABGs per year. The annual upper limit was arbitrarily fixed at 3,000 CABGs. Data points were calculated in 0.25% SSI incidence increments and 125 CABG per year. The cost per patient at each discrete data point was computed with 95.0% confidence over 50 seeded simulation runs with (100%, spECG_[100]_) and without spECG (0% usage, spECG_[0]_). Cost savings per patient were calculated as spECG_[100]_ – spECG_[0]_. The linear regression coefficients were obtained from *Z*_*x,y*_ ~ β_0_ + β_*i*_*X*_*i*_ + σ_*res*_, where *Z*_x,y_ is the delta cost (saving) per patient, β_0_ the intercept, β_i_ the regression coefficient for the *X*_i_ independent variable (SSI incidence and number of CABG), and σ_res_ the residual standard deviation.

## 3. Results

### 3.1. Base case

The economic and clinical outcomes from the base-case simulation are summarized in Table 2. According to NHS Digital Data (23), patients admitted for CABG at Barts Health NHS Trust hospitals had an average LOS of 11.6 days, reduced to 10.7 days without an SSI. Inpatient SSI occurred in 3.96% of cases and added 24.6 days to the LOS; readmission due to SSI occurred in 3.51% of cases and had an average LOS of 12.6 days. The mean base-case simulation cost of care with rECG was GBP 13,096 [95% CI (13,093, 13,099)]. GW was the largest cost contributor, with ECG being the least at 0.015%. The mean LOS was 9.98 [95% CI (9.97, 9.99)] days, closely aligned with input values. The simulation yielded a total of 214 SSI cases, including 202 [95% CI (201, 203)] superficial SSIs and 12 DSWIs [95% CI (12, 12)], compared to the total of 170 cases reported in NHS Digital. The overall incidence of SSIs with rECG was 8.57% (inpatient SSI + readmission due to SSI). The spECG devices reduced the average cost per patient to GBP 12,708 [95% CI (12,698, 12,718)], i.e., a GBP 388 saving [95% CI (−398, −378)] when compared to rECG. As with rECG, the GW was the largest contributor to costs with spECG, and in this setup, ECG monitoring accounted for 0.10% of care costs due to higher spECG procurement costs. The mean LOS was similar to rECG (9.98 vs. 9.82 days). The simulation resulted in 161 superficial and 10 deep sternal SSIs: −51 superficial [−25.25%, 95% CI (−53, −49)] and −2 DSWI [-16.67%, 95% CI (−2, −2)] compared to rECG. The overall incidence of SSIs with spECG was 6.92%.

**Table 2:** Model outcomes from the base-case simulation.

	**rECG, point estimate (95% CI)**	**spECG, point estimate (95% CI)**	**Difference, point estimate (95% CI)**
**Economic outcomes**			
ECG monitoring, £	4,361 [4,356, 4,366]	28,115 [28,082, 28,148]	23,754 [23,748, 23,760]
Surgery, £	3,037,908 [3,034,336, 3,041,480]	3,043,959 [3,041,573, 3,046,345]	6,051 [2,479, 9,623]
ICU, £	10,818,640 [10,801,678, 10,835,602]	10,172,291 [10,168,304, 10,176,278]	−646,349 [−663,311, −629,387]
GW, £	14,701,963 [14,684,675, 14,719,251]	14,472,018 [14,460,673, 14,483,363]	−229,945 [−247,233, −212,657]
Outpatient, £	26,013 [25,962, 26,064]	25,783 [25,732, 25,834]	−230 [−281, −179]
Cost per patient, £	13,096 [13,093, 13,099]	12,708 [12,698, 12,718]	−388 [−398, −378]
Total cost, £	28,588,895 [28,577,689, 28,600,101]	27,742,175 [27,720,427, 27,763,923]	−846,720 [−857,926, −835,514]
**Consequence outcomes**			
Superficial SSI, *n*	202.0 [200.6, 203.4]	151.0 [149.6, 152.4]	−51.0 [−52.5, −49.5]
DSWI, *n*	12.0 [11.7, 12.3]	10.0 [9.8, 10.2]	−2.0 [−2.4, −1.6]
SSI incidence, %	9.25 [9.18, 9.32]	6.92 [6.86, 6.98]	−2.0 [−2.5, −2.2]
ICU, days	2,663 [2,660.9, 2,665.1]	2,620 [2,618.0, 2,622.0]	−43.0 [−45.2, −40.8]
GW, days	21,786 [21,768.9, 21,803.1]	21,437 [21,420.2, 21,453.8]	−349.0 [−366.2, −331.8]
ICU, days/patient	1.22 [1.22, 1.22]	1.20 [1.20, 1.20]	−0.02 [−0.04, 0.00]
GW days/patient	9.98 [9.97, 9.9]	9.82 [9.81, 9.83]	−0.16 [−0.21, −0.11]

Costs are given in 2021 GBP, £, at 95% CI (rounded at the nearest integer, where applicable), confidence interval; ICU, intensive care unit; GW, general ward; SSI, surgical site infection; DSWI, deep sternal wound infection; SOC, standard of care.

### 3.2. Sensitivity analysis

A semi-probabilistic one-way sensitivity analysis was used to examine the robustness of the model to changes in all input parameters (Figure 3). In accordance with the base-case results, the model is predominantly sensitive to changes in GW cost. Increasing GW costs provide an extra 2.54% savings advantage to spECG over rECG. Increasing ICU and surgical costs have limited consequences (<0.5%), whereas PACU cost and time, device procurement, MV cost, and operative time have no bearing on the modeled savings.

The modeled spECR-related savings was tested as a function of SSI incidence (between 1 and 8%) and the number of CABG per year (Figure 4A). A strong dominance of incidence emerged as the driving variable for per-patient savings, while the facility size in terms of yearly CABG is marginal. In fact, NHS facilities (red triangle) far smaller than the base case (green dot) are projected to achieve analogous cost savings, assuming they operate within the same SSI incidence range. Three smaller cardiac units encompassing a large interval of annual CABGs may virtually realize even greater savings per patient than the much larger base case. The contribution of SSI incidence can be estimated at an additional 85 GBP per percentage point of SSI (Figure 4B). In contrast, changes in the number of procedures have a minor and non-significative budget impact of ~8 GBP (*p*-value 0.63) per increment of a thousand CABGs per year (data not shown).

## 4. Discussion

While a randomized clinical trial or other empirical studies would undoubtedly be more conclusive in informing decision-makers and healthcare professionals, these require extensive, lengthy, and expensive data collection, made unpracticable by the COVID-19 outbreak and the consequent strain on ICUs. In addition, limited or non-existent data regarding spECG in the NHS Digital database at the time of writing drove the choice of modeling, an established and practical alternative for simulating hypothetical scenarios with a reasonable degree of approximation in the optic of a preliminary assessment of the technology's potentiality on costs and outcomes.

Drawing on the NICOR's National Adult Cardiac Survey Audit (NACSA) 2021 report (8) and the current literature on the soundness of spECG technology in perioperative cardiac prophylaxis, this model offers an initial assessment of the potential impact on NHS cardiac units. Beyond relevant clinical arguments for improved patient safety addressed in the literature, our model suggests that disposable spECG devices can yield base-case budgetary benefits of about GBP 388 per patient, 95% CI (−398, −378). Savings are driven by a 25 and 17% reduction in the incidence of superficial and deep sternal-wound infections, respectively, compared to rECG. Cutbacks in ICU (6.0%) and GW (1.5%) costs were the primary determinants in consequence of reduced LOS [−1.6% or −0.02 days/patients in ICU −95% CI (−0.04, 0.00), and −1.6% or 0.16 days/patient on GW, 95% CI (−0.21, −0.11)]. Accordingly, the model proved most susceptible to GW and ICU costs in the sensitivity analysis while only marginally affected by other variables. Notable is the preponderant impact of the incidence rate of SSIs on the expected savings with respect to the number of CABG procedures. Variations in SSI by a percentage point predict tangible budgetary shifts, while leaps in the thousands of patients per year yield only marginal gains. This circumvents the assumption that a critical mass of CABG procedures would primarily determine the break-even point for a cost-effective adoption of spECR. Provided they operate within the same SSI incidence interval, relatively small cardiac units can expect relative savings comparable to considerably larger settings. In this respect, consistent with the conclusions from NACSA 2021 (8), it is pertinent to note how small and medium-sized hospitals are most prone to imprecise, underestimated SSI rates amid fragmentary and unexhaustive literature concerning the extent of rECG-related infections (11, 18). The significance of spECG is to be contextualized as part of a bundle of synergistic SSI control measures (e.g., perioperative hygiene programs, wound care, antibiotic prophylaxis, etc.), cost-effective across diverse surgical specialities (37–42).

The anatomy of CABG forces ECG leads and cables in close proximity to sternal wounds, inherently exposing patients to avoidable and potentially fatal complications (13–15). Infection prevention is crucial as durable non-antibiotic prophylactic interventions are becoming increasingly valuable amid grim prospects for nosocomial antibiotic resistance (43, 44). On the other hand, single-use devices in OR operations represent a significant source of hospital waste, disposal costs, and environmental impact (45). Therefore, targeting disposable devices at high-risk procedures is a reasonable compromise to safeguard patients' safety and intervention sustainability.

The reader should be wary of direct extrapolation to other geographies or settings and consider these results within the model's limitations. Nevertheless, conceived with a modular structure from its outset, the model is readily transposable to different scenarios and malleable to implement parameters for the minute simulation of any specific healthcare setup. Altogether, spECGs prospect improved prophylaxis in complex cardiothoracic surgery scenarios along with significant monetary benefits within the NHS setting. Although the model reasonably describes CABG's surgical and postoperative course, some limitations persist. The model's tendency to slightly overestimate the cumulative incidence of infection can be ascribed to two possible reasons. On the one hand, the source data from NHS Digital (23) is rounded to the nearest five, introducing a non-trivial approximation to figures from smaller facilities. On the other hand, in terms of the model, the simulated incidence may be distorted by the BHIS infection risk assignment system. In fact, at this stage, no correlation matrix between the characteristics of the patients could be implemented in the model, and some of the patients entering the simulation may have been assigned unrealistic combinations of characteristics. However, this effect should cancel out in purely probabilistic terms due to the equally likely assignment of under and overestimated BHIS. At last, while the model and sensitivity analysis encompass both first and second-order uncertainty, the OWSA may certainly fail in capturing interactions between input variables, the impact of extreme values or outliers in the input distributions, and assumes that the input distributions are independent, which may not always be the case in practice. However, in this specific case, the model is sensitive primarily to cost inputs and no joint interactions between these inputs can be assumed. In addition, since costs are calculated ex-post to population outcomes, cost inputs have no cross-interactions with other input parameters to affect simulated patient outcomes.

## 5. Conclusions

Based on our analysis, cost savings from reduced SSI incidence are expected to outweigh the additional procurement cost of spECG. As such, spECG has the potential to offer hospitals performing CABG a beneficial alternative to reusable ECG cables and lead wires, both in terms of enhanced patient safety and resource allocation.

## Data availability statement

The original contributions presented in the study are included in the article/Supplementary material, further inquiries can be directed to the corresponding author.

## Author contributions

RS contributed to the conceptualization of the study, design of the model, implementation of the model, collection of input data, interpretation of results, and manuscript writing. MC contributed to the model's implementation, formal data analysis, data visualization, interpretation of the results, and manuscript writing. PS contributed to the conceptualization of the study, clinical expertise, development of the model, and the editing of the manuscript. All authors contributed substantially to the research and read and approved the manuscript.
